# Next Generation Sequencing, and Development of a Pipeline as a Tool for the Detection and Discovery of Citrus Pathogens to Facilitate Safer Germplasm Exchange

**DOI:** 10.3390/plants13030411

**Published:** 2024-01-30

**Authors:** Manjunath Keremane, Khushwant Singh, Chandrika Ramadugu, Robert R. Krueger, Todd H. Skaggs

**Affiliations:** 1 USDA ARS, National Clonal Germplasm Repository for Citrus and Dates, Riverside, CA 92507, USA; manjunath.keremane@ars.usda.gov; 2 Department of Botany and Plant Sciences, University of California Riverside, Riverside, CA 92521, USA; khushwas@ucr.edu; 3 USDA ARS, U.S. Salinity Laboratory, Riverside, CA 92507, USA; todd.skaggs@usda.gov

**Keywords:** citrus germplasm, budwood certification, Next-Generation Sequencing, pathogen testing, biological indexing, real time PCR, huanglongbing

## Abstract

Citrus is affected by many diseases, and hence, the movement of citrus propagative materials is highly regulated in the USA. Currently used regulatory pathogen detection methods include biological and laboratory-based technologies, which are time-consuming, expensive, and have many limitations. There is an urgent need to develop alternate, rapid, economical, and reliable testing methods for safe germplasm exchange. Citrus huanglongbing (HLB) has devastated citrus industries leading to an increased need for germplasm exchanges between citrus growing regions for evaluating many potentially valuable hybrids for both HLB resistance and multilocational performance. In the present study, Next-Generation Sequencing (NGS) methods were used to sequence the transcriptomes of 21 test samples, including 15 well-characterized pathogen-positive plants. A workflow was designed in the CLC Genomics Workbench software, v 21.0.5 for bioinformatics analysis of the sequence data for the detection of pathogens. NGS was rapid and found to be a valuable technique for the detection of viral and bacterial pathogens, and for the discovery of new citrus viruses, complementary to the existing array of biological and laboratory assays. Using NGS methods, we detected beet western yellows virus, a newly reported citrus virus, and a variant of the citrus yellow vein-associated virus associated with the “fatal yellows” disease.

## 1. Introduction

Citrus is a clonally propagated tree crop, and the economic impact of distributing plant material from infected sources can be colossal. The importance of disease-free plant material for the success of the citrus industry was realized early in California starting with the Psorosis Freedom Program in 1937, which later evolved into the Citrus Variety Improvement Program in 1958, and was renamed the Citrus Clonal Protection Program in 1977 [1]. Early research at the University of California by Wallace and others led to an understanding of how diseases like psorosis and tristeza spread through infected budwood. Growers and nurserymen from California requested the University to assume responsibility for maintaining healthy plants in primary foundation blocks to provide the citrus industry with disease-free and true-to-type budwood under the regulatory control of the California Department of Food and Agriculture (CDFA). Biological indexing was the predominant technique used in the early budwood certification programs [2].

Citrus quarantine, certification, and distribution programs are designed to deliver healthy plant materials for both researchers and growers. The effectiveness of these programs depends on the tools used for pathogen detection. The efficacy of techniques used determines the success of safe germplasm movement. Initially, the detection of psorosis and tristeza by graft inoculation on indicator plants was used for the selection of virus-free mother trees. Additional indicator plants were included for indexing as knowledge about other diseases increased. Serological and molecular methods were incorporated as essential tools as they became available. Molecular methods such as real-time PCR are valuable and amenable to high throughput assays but allow for testing of only known pathogens. A limitation of the commonly used lab-based methods is that non-target pathogens are not discovered. Because of these factors, the California budwood certification program has retained the biological indexing methods, as have many other citrus budwood certification, sanitation, and introduction programs.

In recent times, several citrus diseases have threatened the survival of the industry. Hence, there is an increased interest in screening new breeding materials in different citrus growing areas for disease resistance and horticultural performance. The development of rapid and reliable budwood testing methods will facilitate the movement of certified plant materials across state and international borders. In recent years, metagenomic approaches have been used for the detection of pathogens because of advances in Next-Generation Sequencing (NGS) technologies. NGS, also referred to as High-Throughput Sequencing (HTS) or deep sequencing, can detect multiple known pathogens as well as novel and emergent pathogens, and hence appears to be the technique of choice for budwood certification.

Both biological and laboratory-based methods of pathogen detection have several limitations. Biological indexing is labor-intensive and time-consuming, and not amenable to high throughput processing. Changes in greenhouse conditions, grafting efficiency, soil, fertilizers, light, temperature, etc., can affect the success of biological indexing [2,3]. Many viruses and/or virus populations can be non-symptomatic on standard indicator plants. Pathogen detection by serological and molecular methods is target-specific; the techniques may not detect pathogen variants that have sequence differences in the genomic regions selected for designing the detection assay. NGS methodology may be able to circumvent most of the problems encountered with both biological and laboratory-based detection methods.

NGS technology allows for the identification of most viruses in a sample without prior information about the pathogen status [4]. NGS has the potential to detect the nucleic acids of any organism present in a sample [5,6,7]. Scientists from the Animal and Plant Health Inspection Service (APHIS), the regulatory agency of USDA, collaborated with scientists from multiple countries and have developed guidelines for the use of NGS technologies to detect plant pathogens and pests [8].

Plant virus detection and discovery using NGS were described in detail [9]. NGS has been used for the detection of several new viruses [10,11,12,13]. In citrus, NGS has also been used for the discovery and characterization of various novel viruses including Citrus chlorotic dwarf-associated virus [14], citrus yellow vein clearing virus [15], citrus leprosis virus cytoplasmic type 2 [16], citrus vein enation virus [17], citrus tatter leaf virus [18], and citrus yellow vein virus [19]. NGS-based microRNA analysis was used to analyze populations of Citrus dwarfing viroid [20]. NGS data was also used to develop e-probes for multiple citrus pathogen detection [21]. Various bioinformatics pipelines have been developed for the identification of viral sequences from NGS data including VirFind [22] and Virusdetect [23]. Other pipelines available are summarized in a review by Villamor et al. [9].

In clonally propagated crops like citrus, the economic consequences of the use of infected budwood can be severe. Citrus concave gum disease has been considered a disease of unknown etiology since 1930s. No vectors are known, and the disease is believed to be transmitted mostly through infected budwood. In the absence of molecular characterization of many viruses, certification programs depended mostly on biological indexing for pathogen detection; development of symptoms for diseases like concave gum is dependent on growth conditions during indexing. Disease symptoms on inoculated indicator plants may disappear quickly leading to inconclusive results. The widespread distribution of certain citrus diseases throughout the world may be attributed to the use of infected budwood. Recently, the NGS analysis of trees with citrus concave gum has led to the discovery of two negative stranded coguviruses, citrus concave gum-associated virus (CCGaV) and citrus virus A (CiVA) [24,25].

Adaptation of NGS as a tool for the certification of citrus appears to be highly useful. NGS analysis can detect both known and novel pathogens. Proper guidelines need to be developed before NGS can be used as a certification tool. There is an effort to integrate NGS as an important tool for certification in South Africa [26,27]. In the present study, 21 plant samples, including fifteen from the pathogen-positive inventory of the USDA National Clonal Germplasm Repository for Citrus & Dates, Riverside, California, USA, were analyzed by NGS transcriptome study. RNA was used as the target for the detection of pathogens with both RNA and DNA genomes (viruses and bacteria). First, we depleted the sample of ribosomal RNAs, constructed NGS transcriptome libraries, and sequenced them on the Illumina platform. We developed a workflow in the CLC software, v 21.0.5 environment to analyze NGS transcriptome data, removed host sequences, and constructed de novo assemblies of non-host sequence reads. Three local databases were generated using: (i) all available sequences of major citrus viruses, (ii) representative genomes of all viruses, and (iii) genomes of known citrus bacterial pathogens. Local blast searches for all viruses and citrus bacterial pathogens were carried out. The results from NGS-based pathogen detections were compared to existing data developed using available biological and laboratory-based test results. We report the detection of multiple citrus viruses and bacteria from the metatranscriptomes of several samples. Raw reads from all the samples analyzed have been deposited in NCBI and are freely accessible for further analysis, as needed. Unlike other biological and laboratory detection methods, the raw data and results from NGS analysis can be made publicly available, making this an open process.

## 2. Materials and Methods

### 2.1. Plant Samples

A total of 21 plant samples were used in this study. Four plants (samples 1–4; Table 1) used for the study belonged to genera closely related to *Citrus*, and *Poncirus trifoliata,* held in quarantine at the USDA Repository. A healthy plant (*C. sinensis*) from the greenhouse (sample 10) and a *C. sinensis* plant graft inoculated with *Candidatus* Liberibacter asiaticus from a Biological Safety level 3 facility (sample 11) were included in the analysis. Fourteen samples were selected from the pathogen-positive inventory.

Sample collection and further processing were done in two sets: samples 1–12 in the first set, samples 13–21, and repeat runs of 5–7 in the second set. Three of the eight libraries (#15, 16, and 19; Table 1) were processed using combinations of samples as shown in Table 1. Further information on each sample can be found in the hyperlinks provided in Table 1. Most of the isolates in the pathogen-positive inventory were collected over the last 50–60 years and are maintained in quarantine facilities. The known disease status of all 20 plant samples used in the study is indicated in Table 1.

### 2.2. Sample Processing and Library Construction

Total RNA was extracted from plant tissue (Table 1) using Qiagen Plant RNeasy kits (Qiagen, Inc., Germantown, MD, USA), including on-column DNase reaction, according to the manufacturer’s protocol. Extracted RNA samples were stored at −80 °C. Libraries were prepared using the Zymo-Seq Ribofree Total RNA Library Kit (Zymo Research, Irvine, CA, USA) according to the manufacturer’s instructions. Briefly, about 0.5 to 1 µg of total RNA with an A_260_/A_280_ ratio of >1.8 was used for reverse transcription. The cDNAs were processed for “Ribofree^®^ Universal Depletion” to remove ribosomal RNAs. The cDNAs were ligated with P7 adapters, and the second strand synthesis was carried out. The double-stranded DNA from the above reaction was ligated with P5 adapters and a short PCR reaction of 12 cycles was carried out with unique dual-index primers (barcoding). The reactants between each of the above steps, and after the final step were cleaned up using “Select-a-size Magbeads”. The final elution of the library was carried out in 25 µL of DNA elution buffer and stored at −20 °C. The first set of 12 libraries was shipped for sequencing to Zymo Research, Irvine, CA, USA, and the second set of eight samples to Quick Biology Inc., Monrovia, CA, USA. The pooled libraries were sequenced to generate 100 bp paired-end (PE) sequences (#1–12) or 150 bp PE sequences (#13–20) using the HiSeq platform (Illumina, San Diego, CA, USA).

### 2.3. Workflow for Bioinformatics Analysis

**Figure 1:**
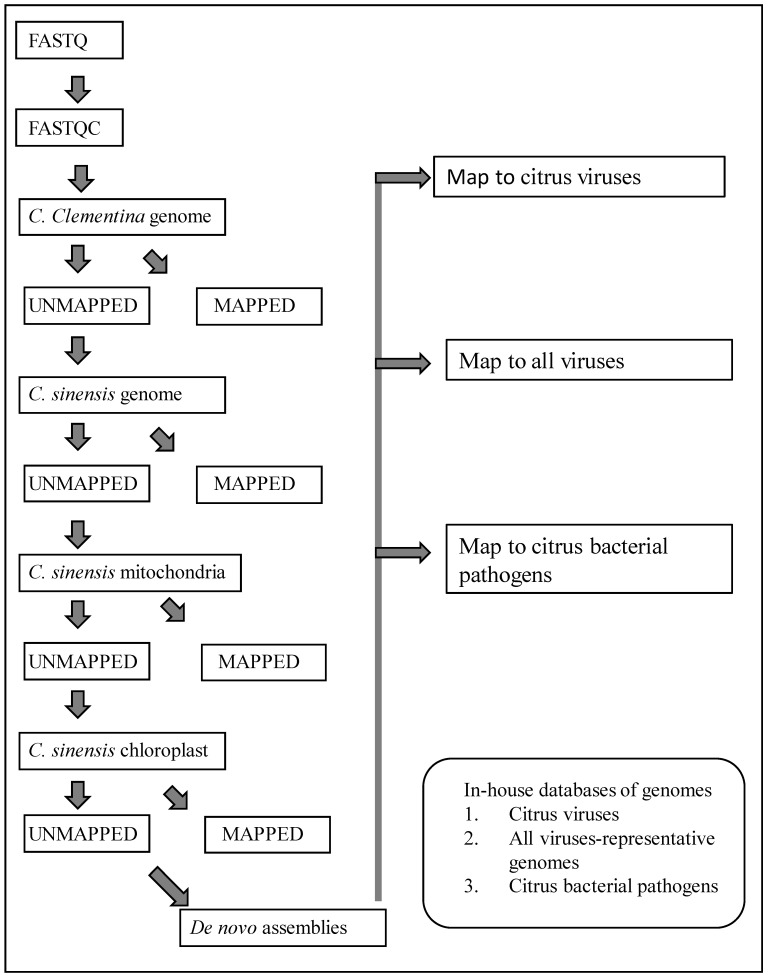
Workflow in CLC Genomics Workbench software used for removal of host sequences, generating de novo contigs of unmapped sequence reads, and local blast for detection of citrus viruses and bacteria.

Data processing was done using a custom workflow (Figure 1) created in CLC Genomics Workbench software (Qiagen Inc., Germantown, MD, USA). Sequence reads were trimmed to remove poor-quality sequences. The remaining high-quality sequences were mapped successively to the genomic sequences of *C. clementina* (GCF_000493195.1), *C. sinensis* (GCF_022201045.2), and chloroplast (DQ864733) and mitochondrial (NC_037463) sequences of *C. sinensis*. At each of the above mapping steps, only the unmapped sequences were used for the next step. The unaligned sequence reads at the end of the mapping steps were used to assemble de novo contigs.

### 2.4. Pathogen Detection

Three in-house databases were created in the CLC environment as described below by importing from NCBI: (i) all available full-length genomes of citrus viruses of interest in the present study; (ii) representative genomes of all viruses from the RefSeq database; and (iii) complete genomes of all available citrus bacterial pathogens. A local blast search of de novo contigs of non-host sequence reads was conducted sequentially using all three pathogen genome databases. Where necessary, multiple contigs of some viruses were combined by re-mapping the reads to the closest viral genome as a reference. In the case of DNA pathogens (only bacterial pathogens included in this study), a blast search of multiple contigs was used to identify the pathogen.

Beet western yellows virus (BWYV) sequences from citrus were analyzed using blast search to find closely related poleroviruses. Multiple sequence alignment of nucleotide sequences was performed as described [28]. ClustalX v2.0 [29] was implemented with default parameters, and phylogenetic trees were generated using ClustalX and MEGA 11.0 [30] applying the neighbor-joining method [31]. The reliability of branches was inferred from bootstrap analysis of 1000 replicates. The final phylogenetic tree was then edited by using ITOL (http://itol.embl.de/, accessed on 12 December 2023). Recombination analysis of BWYV isolates was performed using various algorithms included in the RDP software package v4.0 [32].

Citrus yellow vein-associated virus (CYVaV) genome sequences identified in this study were compared to other genomes in the NCBI database using blast search. The 5′ and 3′ untranslated regions and two open reading frames were compared to respective regions of other viruses with similar genome organization.

The sequence of Citrus blight-associated pararetrovirus (CBaPRV) identified from the analysis of the de novo assembly from ‘Fuming Evergreen’ trifoliate (*Poncirus polyandra*; sample #21 in library #19) was used to do an NCBI blast search. A real-time PCR assay was developed to specifically detect the endogenous CBaPRV reportedly expressed at higher levels in roots with citrus blight, but not other citrus endogenous pararetroviruses (CEPRV) (see next section). These CEPRVs have been reported to lack the 3′ terminus of about 2000 bases of the 7 kb viral genome. Expression of CBaPRV of leaves and roots of ‘US 942′ rootstock seedlings grown from seeds received from Florida was assayed by real-time PCR. Further, over 1000 plants of various genotypes of citrus from greenhouse and field were tested by qPCR for expression of CBaPRV. Blast searches of genomes of several members of the subtribe Citreae and Clauseneae were conducted to detect CBaPRV. The negative test results from non-trifoliate accessions may also be due to sequence differences in the target region selected for real-time PCR assay (Supplementary Table S3).

### 2.5. Real-Time PCR Assays

Real-time PCR primers were designed for the host reference gene and four viruses of interest: Beet western yellows virus (BWYV), citrus yellow vein-associated virus (CYVaV), citrus virus A (CIVA), and citrus blight-associated pararetrovirus (CBaPRV). To specifically amplify the virus reported to be associated with citrus blight, and to avoid amplification of the other known citrus endogenous pararetroviruses, a region of about 6 kb in the genome was selected as the PCR template. For the other three viruses, primers were designed to amplify conserved regions of available genomes. As a reference, the malate dehydrogenase gene [33] was used, except that the forward primer was designed to incorporate 21 bases from the 3′ end of exon 12 and four bases from the 5′ end of exon 13 so that the amplification would take place only from the reverse transcribed RNA template, and not from genomic DNA.

Synthetic DNA (gBlocks^TM^) was synthesized for the above five templates by Integrated DNA Technologies, Inc., San Diego, CA, USA. Serial dilutions were made from 10^8^ to 10^0^ copies/µL and used for real-time PCR assays using appropriate primers and probes. Standard curves were constructed to calculate linearity and reaction efficiency.

Complementary DNA (cDNA) was synthesized using a mixture of 5 µL of RNA, 2 µL of Lunascript (New England Labs, Ipswich, MA, USA), and 3 µL of water, and incubating at 25 °C for 2 min, 55 °C for 10 min, and 95 °C for 1 min, and then cooled to room temperature. Real-time PCR mixes were prepared using 10 µL of Sso Advanced universal probes supermix (Biorad, Inc., Hercules, CA, USA), 240 nM primers and 120 nM probe for both the target virus and MDH, and water to make up the volume to 20 µL. PCR reactions were conducted in 96 well plates in a ViiA7 real-time PCR machine using the thermal profile mentioned here: 95 °C for 20 s, 40 cycles of 95 °C for 5 s, and 60 °C for 20 s. Both dilute gBlocks^TM^ and known positive extractions served as positive controls. RNA extractions were also made from 40 seedlings of US-942. All extractions were assayed for the presence of the four viruses mentioned above by real-time PCR.

### 2.6. Testing Inventory of Pathogen-Positive Plants

The citrus germplasm repository has over 115 accessions of citrus pathogens, maintained in planta in quarantine with appropriate permits. While most of them were collected from within California over the last 50 to 60 years, others were received from other states or countries under appropriate permits. Real-time assays for the presence of BWYV, CIVA, CYVaV, and CIVA were conducted.

## 3. Results

### 3.1. Bioinformatics Analysis of NGS Data

Twenty RNA-Seq libraries from 21 plant samples (Table 1) constructed in the present study were checked for library quality by the TapeStation system (Agilent Technologies, Santa Clara, CA, USA). The number of reads and read lengths for each library used in the study are shown in Table 2 along with links to metadata and raw data submitted to the National Center for Biotechnology Information (NCBI) databases. With the first set of 12 samples, an average of about 30 million reads (100 bases per read) were generated per sample. In the second set of eight samples, about 120 million reads (150 bases per read) were obtained per sample (Table 1).

The workflow used for bioinformatics analysis is shown in Figure 1. The sequence reads were trimmed to remove poor-quality sequences. Over 96% of the good-quality reads were retained in all samples. The exact number of host genome sequences removed at each step is shown in Table 2. About 81% to 98% of the sequence reads were mapped to host genomes (Table 2). The number of unmapped sequence reads varied significantly between the samples ranging from 1.17% to 18.07%. Sample 6, with 13.27% of the total sequence reads unmapped to the host genome was infested with thrips; sample 20 with 18.07% of sequence reads unmapped to the host genome was infested with mealybugs. A sample from the citrus relative *Clausena* spp. showed a higher percentage of sequence reads that did not map to the citrus genome probably because of sequence diversity between *Citrus* and *Clausena* (Table 2). Many unmapped sequence reads in the current study presumably originated from the associated insect species.

Local BLASTN search of de novo assemblies generated from unmapped reads was conducted against a database of citrus viruses and bacterial pathogens. The pathogens described in this study are listed in Table 3. The analysis resulted in the identification of many citrus pathogens and other citrus-associated viruses (Table 4). No citrus pathogens were identified in samples 1–4 and 10. Samples 8, 11, and 12 were infected by a single citrus pathogen while others had mixed infections with multiple pathogens. Mapping of sequence reads to the reference genomes was carried out to calculate the depth of the genome coverage of the pathogen (Table 4) which ranged from about 280,000× (for RNA3 of both CLRV and CVV in library #15) to below 10× for some viruses.

### 3.2. Detection of Pathogen Sequences

The current nomenclature of viruses and bacteria is used throughout the manuscript. Previously used names mentioned in old literature, and maintained in database records are relevant. For clarity, the current nomenclature of viruses and bacteria along with alternate names used in the literature are provided in Table 3.

Table 5 compares the results of pathogen testing in the present study with previous characterization using several biological and laboratory-based assays. Beet western yellows virus (BWYV) was detected in samples 5 (libraries 5 and 17), and 16 (library 16). This is the first report of BWYV from citrus. A variant of Citrus yellow vein-associated virus was found in the plant with “fatal yellows”, a disease of unknown etiology.

### 3.3. Insect Viruses and Bacteria

Transcriptome analysis also revealed a few viruses were presumably derived from insect pests prevalent in the greenhouses. A picorna-like virus (PLV; GenBank accession MW674792) was detected in sample 6; real-time PCR analysis of citrus trees from the greenhouse and field revealed its widespread presence and uneven distribution within plants and variability during different seasons. Analysis of sequence reads from sample 6 unmapped to the host revealed the DNA of a common greenhouse pest, *Scirtothrips citri.* Analysis of unmapped sequence reads from sample 20 revealed the presence of another greenhouse citrus pest, mealybug, *Planococcus* sp. Complete genomes of two endophytic bacteria of *Planococcus*, *Candidatus* Trembalya princeps (*Ca*TP) and *Candidatus* Monarella endobia (*Ca*ME) were also recovered from sample 20.

### 3.4. Suppression of Viruses

Sample 7 (Cristacortis, IVNO 8078) was collected at two different times and two NGS libraries were constructed (#7 and #18, Table 1). Both libraries showed the presence of CBLVd, CBCVd, and CPsV. While all four viroid sequence reads were present in library #7 at about 60× to 548× depth, in library #18 sequence reads belonging to HSVd and CBCVd were either absent or very low; reads of CBLVd were present at over 4200× genome coverage (Table 4). Similarly, both CVd V and BWYV sequence reads increased significantly in library #17 compared to library #5. However, it did not appear to affect the CDVd population (Table 4).

For most of the viral sequences identified in the present study, full-length genome sequences were recovered. A total of 46 full-length genome sequences have been deposited in NCBI (Table 6).

### 3.5. Analysis of Samples Infected with “Fatal Yellows” Disease

The pathogen sequences identified from “fatal yellows disease” (library 14, Table 1), a disease of unknown etiology, contained sequences of CYVaV along with very low levels of CLRV and CVV (Table 4). The genomes of CYVaV from fatal yellows (Table 6; MZ330113) and citrus yellow vein (Table 6; accession MZ33089) differed significantly (Table 7) showing only about 86% nucleotide identity at the genome level, 91% at the amino acid level (between the two RNA dependent RNA polymerase), and 86% identity with the hypothetical protein.

### 3.6. Analysis of BWYV Sequences

**Figure 2:**
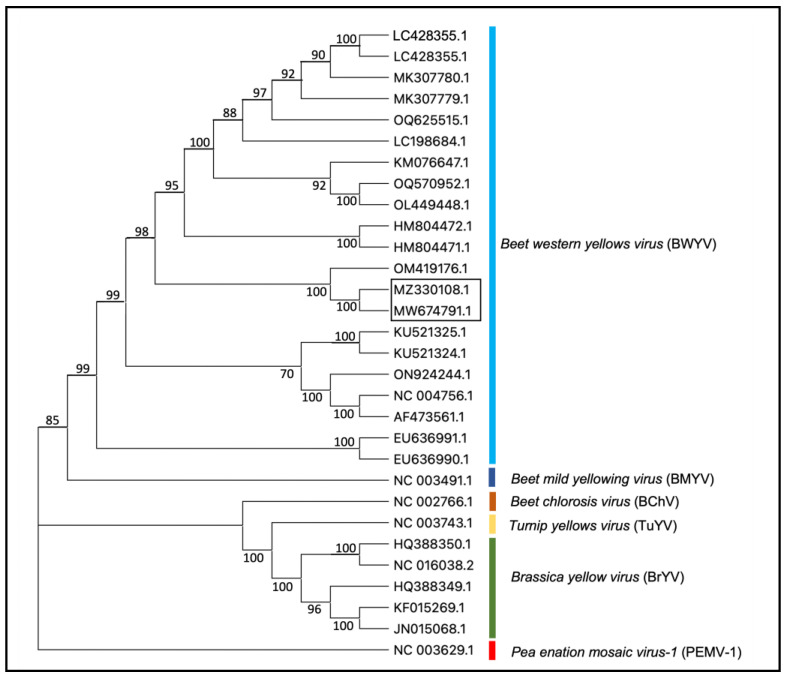
Phylogenetic tree representing the relationship between BWYV isolates and other closely related viruses in the genus Polerovirus. Accessions from the citrus described in the present study are shown in the box. Pea enation mosaic virus-1 (PEMV-1, genus Enamovirus, NC_003629) is used as an outgroup. The phylogenetic tree was constructed using the neighbor-joining method and visualized with MEGA11.0. Bootstrap values shown at the nodes indicate data analyzed from 1000 replications. The names of different viruses and accession numbers of isolates used in this study are shown.

The blast search of de novo contigs of non-host sequence reads from two plants (IVNO 4501 and 5046) revealed the presence of BWYV in these samples. The sequences of two BWYV genomes reported in this study are about 98.5% identical (Supplementary Table S1). BWYV isolates from citrus showed 97% nt identity with an isolate of BWYV reported from *Coriandrum sativum* from Cyprus (OM419176.1) [34]. Other BWYV genomes included in this analysis showed about 81–93% identity with the citrus isolates; 93% nt identity with SDWF16 isolate (MK307780.1) on *Capsicum annuum* from China, NJ22 isolate on *Raphanus sativus* from S. Korea (OQ625515.1), and S19 isolate reported on spinach from Japan (LC428356.1) [35]. Phylogenetic analysis of BWYV and a few closely related poleroviruses (Figure 2) indicated that the BWYV genomes from citrus clustered closely with other BWYV genomes. A similar analysis using BWYV genome sequences from different geographic regions (Supplementary Figure S1) shows that the citrus isolate of BYWV clusters closely with an isolate from coriander reported from Cyprus and some other isolates from Asia.

**Figure 3:**
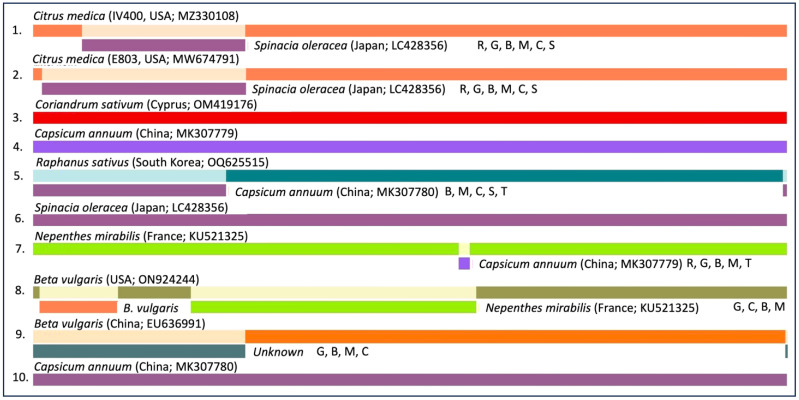
Recombination analysis of Beet western yellows virus (BWYV) sequences from various host plants. Analyses were performed using various algorithms included in the RDP software package [32]. The order of the designation of the recombination events is as follows: host plant name, country of origin, NCBI accession number, and algorithm (R, RDP; G, GENECONV; C, Chimaera; M, MaxChi; B, Bootscan; S, SiScan; T, 3SEQ). Only recombination events with a *p*-value of <0.01 detected based on at least three different algorithms are shown.

Recombination analysis using the RDP software package [32] strongly supported a recombination event at the 5′ region of the BWYV/citrus genome with an isolate of BWYV on spinach from Japan (Figure 3). These preliminary results suggest that BWYV infected citrus in Asia well before it was introduced to Western countries. However, further analysis of citrus trees from other geographic regions may help understand the origin of BWYV in citrus.

### 3.7. Analysis of Citrus Blight-Associated Pararetrovirus Sequences

**Figure 4:**
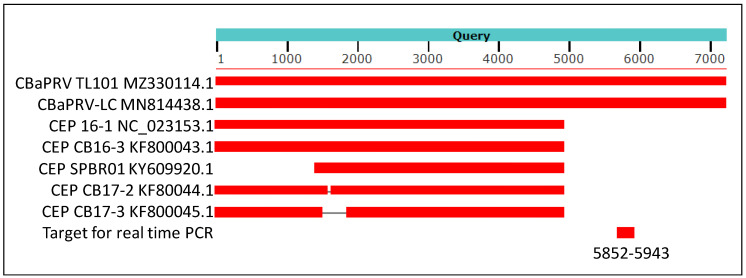
A cartoon showing results of blast analysis of 7652 nucleotides long assembly (NCBI accession MZ330114.1) of sequence reads from library 19 with IVNOs 2327 and 3591. The above assembly was created by conducting a de novo assembly of sequence reads unmapped to the citrus genome (as described in Figure 1). The sequences of Citrus blight-associated pararetrovirus (CBaPRV-LC; NCBI accession MN8814438.1) showed about 92% nucleotide identity with the query sequence while five other sequences in Genbank showed about 90% identity at the nucleotide level, restricted to the 5′ 5000 bases of the viral genome. The genomic location utilized to design a real-time PCR assay is indicated (nucleotides 5852–5943).

The genomic sequence of citrus blight-associated pararetrovirus (CBaPRV) showed 92% nucleotide identity, with genomic sequences of the CBaPRV-LC isolate reported to be associated with citrus blight. Several other endogenous pararetrovirus-like sequences also showed very high similarity, but none were of genomic length (Figure 4).

### 3.8. Real-Time PCR Analysis for Detection of New Citrus Viruses

**Figure 5:**
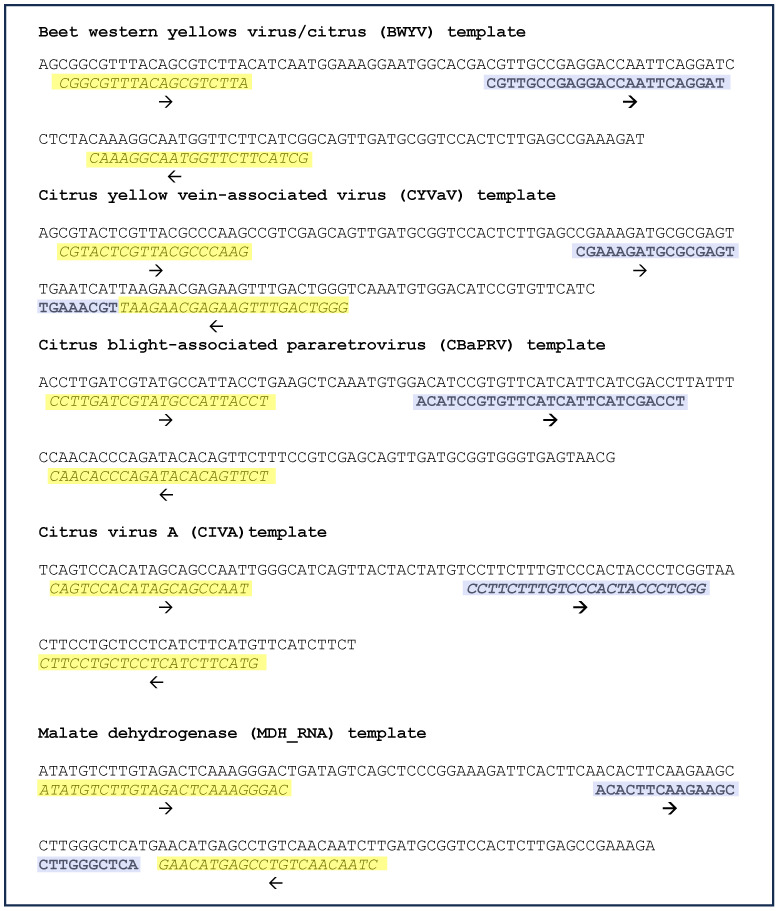
Real-time PCR: templates, primers, and probes. Sequences of synthetic DNA templates for four viruses of interest were discovered in this study and for the reference gene, malate dehydrogenase (RNA sequence) is shown. The forward and reverse primers are highlighted yellow; the probe sequences are highlighted purple and are depicted in bold font. The direction of primers and probes is indicated by an arrow below the sequences. Further details of primer design for specific amplification of RNA, but not DNA are shown in Supplementary Figure S2.

The sequences of synthetic DNA templates (gBlocks^TM^), primers, and probes designed for real-time PCR assay for BWYV, CYVaV, CBaPRV, CiVA, and the host reference gene, MDH, are shown in Figure 5. The primers and probe for selective amplification of MDH RNA, but not DNA was achieved by the design of the forward primers in the exon regions as shown in Supplementary Figure S2.

The gBlocks^TM^ were provided by the manufacturer at 10 ng/µL concentration. Based on the calculated molecular weight, moles/µL and copies/µL were computed. Stock solutions were prepared at 10^8^ copies/µL, and serial dilutions from 10^8^ to 10^0^ copies/µL were used for generating standard curves using appropriate primers and probes. These assays conducted for all five templates (BWYV, CYVaV, CBaPRV, CIVA, and MDH) with corresponding primers and probes revealed calibration curves with a coefficient of correlation (R^2^) between 0.9991 to 1.000. The slope of the curve ranged from −3.38 to −3.57, and the PCR efficiencies were calculated to be 91.4% to 95.7% (Table 8). The Ct values of the three technical replicate runs for each of the five templates (dilutions from 10^2^ to 10^8^) were consistent (±0.35) in the replicate reactions.

Real-time PCR analysis was conducted using plant extracts from the pathogen-positive plant inventory. Plant samples originated from the quarantine facility of the USDA Germplasm Repository; extractions and PCR were carried out to detect four viruses, BWYV, CYVaV, CIVA, and CBaPRV, along with MDH as a reference gene. BWYV was detected in six of the 103 accessions tested. Four of the accessions were collected originally from California, and one each was from Florida and Spain (Table 9). CYVaV was detected in only two accessions, both collected from California. CIVA was identified in six accessions, five originating in California and one from Florida. CBaPRV was not detected in any of the 103 accessions in the positive inventory plants.

DNA and RNA extractions from both leaves and roots of 40 seedlings of rootstock variety ‘US 942’ were positive for CBaPRV. Further analysis of over 1000 plants of various genotypes collected from protected facilities and fields was carried out. Higher levels of expression of CBaPRV appeared to be restricted to trifoliate and trifoliate hybrids (Table 10).

### 3.9. Multiple Copies of CBaPRV Integrated into Citrus Genomes

A blast search of genomes of several members of Aurantioideae revealed that several copies of the full-length genomes of CBaPRV are endogenous in all the nine chromosomes in members of the tribe Citreae analyzed in the study (Supplementary Table S2), but not in the tribe Clauseneae (*Murraya paniculata* and *Bergera koenigii*). The target sequence used for real-time PCR assay from the sequence of CBaPRV (accession MZ330114.1) was 100% identical to the sequence reported to be associated with citrus blight (MN814438.1). However, the target sequences in the five genomes of the members of Aurantioideae showed several nucleotide differences (Supplementary Figure S3).

## 4. Discussion

A major challenge of the currently used biological and laboratory techniques is the difficulty encountered when comparing results from different laboratories. Often this results in duplicating all or many of the biological and laboratory tests in both donor and receiving industries resulting in extended waiting time for both researchers and growers to receive the germplasm of their interest. Novel pathogens and strains are often missed by the currently used testing methodologies. Often disease symptoms may not be expressed on all indicator hosts at all times; it is vital to detect all pathogens in imported propagation materials.

### 4.1. NGS Is Becoming a Method of Choice for Pathogen Detection in Multiple Crop Plants

NGS is a useful technology as an additional tool for the pathogen testing process and may be able to resolve some of the ambiguities that arise with the standard methods. NGS can be a powerful tool since it can detect asymptomatic infections, novel pathogens, and variant populations [3]. NGS can be applied to solve problems in a range of areas involving viral diseases, in determining the etiology of viral diseases, in virus characterization, taxonomy, viral population genetics, and in diagnostics of plant viruses [6]. Raw data on NGS analysis of test plants can be made available to others via public databases so that the data can be re-analyzed by interested groups for detection of the pathogen sequences. This makes the procedure of budwood certification an open process.

A major goal of the present study was to compare the results of NGS-based pathogen detection with the currently used biological and laboratory-based detection methods. The long-term goal is to utilize NGS as an additional tool for pathogen testing so that the process can be quick and more efficient. The USDA Repository in Riverside maintains, under appropriate regulatory permits, over 100 accessions in its pathogen-positive inventory. Many of the isolates were collected by scientists in California over the last 50 to 60 years and their pathogen status has been well studied using both biological and laboratory-based assays. They appeared to be excellent samples for the current study. Since HLB and certain other exotic disease agents are not maintained in this collection, a plant sample infected with HLB-associated CLas was obtained from an approved containment facility.

Viral metagenomics has revolutionized virus discovery using a wide variety of clinical samples [36]. Thousands of novel viruses have been discovered over the last decade through viral metagenomic studies [37]; these methods have been extensively used in the discovery of plant viruses for about 15 years [12,38,39]. Several novel citrus viruses have been discovered through NGS [14,15,16,17,19].

### 4.2. NGS Facilitates the Genome Characterization of Viruses and the Discovery of Both Viral and Bacterial Pathogens including New Citrus Viruses

A comparison of two sets of libraries, one with about 20–30 million reads per sample (libraries 1–12; Table 1) and another with about 120 million reads per sample (libraries 13–20; Table 1) suggested that the data from libraries 1–12 may be adequate for identification of pathogens; increased sequence depth may facilitate recovery of full-length genome sequences and increase the sensitivity of the analysis through detection of pathogens present at low titers.

A comparison of results from the present study on NGS analysis and previous test results (Table 5) shows that by utilizing NGS, we were able to find sequences of all pathogens found previously by biological, serological, and molecular methods. In addition, the method also identified new viruses associated with citrus: for example, the detection of BWYV and a variant of CYVaV. The significance, if any, of BWYV in citrus is currently not known.

Sequencing at higher depths resulted in the identification of some viruses occurring at low levels (Table 4, compare libraries 5 and 17). Except for a small number of viruses with low genome coverage (Table 4), full-length genomes were recovered from the NGS data for most of the viruses detected. A total of 46 of these full genome sequences have been deposited in NCBI (Table 6). The present study also revealed sequences of bacterial transcripts (*C*Las and SC; Table 3). NGS, as deployed here, should be able to detect all pathogen genomes with RNA in their life cycle, provided that the bioinformatics analysis includes the target genomes in the database for blast search. Accordingly, the present study was directed at finding all viruses, and known bacterial pathogens of citrus. Most of the pathogens found in this study also confirmed the results of previous studies (Table 5).

### 4.3. NGS Analysis Discovered New Citrus Viruses

BWYV, not previously reported from citrus, was detected (as six isolates) from plants that originated from various geographic locations. While the two genomic sequences of BWYV from citrus showed about 98.4% similarity, the other closest genome identified was from coriander from Cyprus with about 97% identity at the nucleotide level (Supplementary Table S1). Phylogenetic analyses (Figure 2 and Supplementary Figure S1) of the genomic sequences confirmed the identity as BWYV. Its close relationship with several isolates from Asia and its presence in isolates collected from different geographic regions suggests that BWYV may not be a new introduction to citrus. Recombination analysis suggests that the BWYV/citrus isolate may have resulted from recombination with a genomic fragment from a spinach isolate of BWYV, but the significance of this is not clear. Pathogenicity and interaction with other viruses need to be studied.

**Figure 6:**
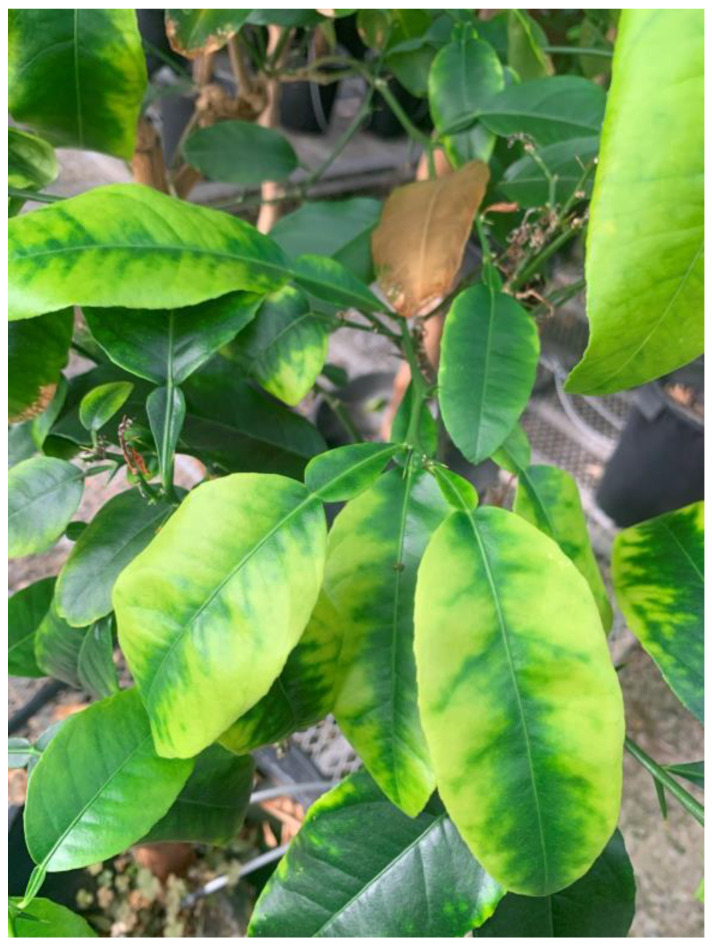
Symptoms of “Fatal yellows” disease on citron (*C. medica*) plants in the greenhouse.

Fatal yellows was reported as a disease of lemons on Alemow rootstock [40]. Typical symptoms of yellowing of both veins and lamina are shown in Figure 6. Corky veins and rubbery stems can also be seen. An isolate has been maintained at the quarantine greenhouses since 1980 as a disease of unknown etiology. Analysis of samples from the plant showing “fatal yellows” (FY) symptoms showed the presence of a variant of CYVaV, which had been reported earlier to be associated with citrus yellow vein disease [19]. Even though the sequences of the FY-associated virus genome varied from the Citrus yellow vein-associated virus by about 14% (Table 7), they are considered the same species because of similar genome organization. The fatal yellows isolate had very low levels of CLRV and CVV, and the yellow vein sample had low levels of CVV and several other viruses (Table 4).

Cristacortis disease was described in 1968 from Corsica on sweet orange trees on sour orange rootstock [41]. Concave gum-like symptoms were observed on both scion and rootstock. It is not clear how to interpret the results from the analysis of plant samples with cristacortis, a disease with unknown etiology. The disease causes vertical depressions in the wood with corresponding pegs on the bark of sour orange and other citrus accessions. This isolate was received from Corsica in 1997 and has been maintained in sweet orange plants. The isolate was associated with four different citrus viroids and CPsV by both NGS analysis and qPCR (Table 4 and Table 5). The populations of HSVd and CDVd varied significantly in samples collected at different times. Since concave gum-associated viruses were not present, and none of the associated pathogens are known to cause cristacortis symptoms, we cannot draw conclusions at this time. The association of viruses in multiple samples with cristacortis may be useful for understanding its etiology.

Real-time PCR (qPCR) tests were developed for four selected pathogens of special interest discovered in this study. A qPCR analysis of over 100 samples was useful in confirming the presence of CPsV and CiVA in the positive pathogen inventory at the USDA Repository. Analysis by qPCR using CYVaV-specific primers and probes detected its presence in only two isolates in the positive inventory (Table 9).

### 4.4. Relevance of Citrus Blight-Associated Pararetrovirus Genome Sequence

The genomic sequence of CBaPRV, which has been reported to be highly expressed in trees with citrus blight disease [42], was detected in one of the samples studied (#21 = RRUT 178 = IVNO 3591). This was of serious concern since blight is a disease that causes significant damage in Florida [43] and several other locations [44,45] but has not yet been reported in California. A real-time PCR assay was developed in this study to selectively detect only CBaPRV, but not other CEPRVs (Figure 4). Greenhouse-grown seedlings of the trifoliate hybrid rootstock ‘US 942’, propagated from seeds received from Florida, were found to have high levels of CBaPRV expression. Assuming that the expression of CBaPRV was associated with citrus blight [42,46], these results were of serious concern. A detailed analysis of the expression of CBaPRV was studied in over 1000 accessions belonging to various genotypes. The high expression level was limited to trifoliates and trifoliate hybrids (Table 10). These results suggest that CBaPRV is constitutively expressed in trifoliate genotypes.

A search for full-length genomic sequence of CBaPRV in several genomes of Auratioideae, a subfamily of Rutaceae to which citrus belongs, revealed that multiple copies of CBaPRV are present in almost all chromosomes of the genomes of members of the tribe Citreae analyzed in the study (Table S2), but not in members of the tribe Clausineae. Multiple copies of full-length or near full-length genomes of CBaPRV were found in most genomes analyzed. The presence of CBaPRV in citrus may not be diagnostic of citrus blight.

### 4.5. Detection of Viruses and Bacteria from Insect Pests

Library #6 (P215) yielded unexpected results since an unknown picorna virus (MW674792) was found. Similar results were reported in metagenomes of honeybees collected from citrus-growing regions in Australia (MG995731 and MG995724). Identical results were obtained upon repeating the sequencing of the same library, but not when a new library (#17) from the same plant sample was sequenced at a different time. Real-time PCR analysis of several samples showed the presence of the picorna virus in greenhouse-grown seedlings and several other plants in both the greenhouse and field. The results strongly suggested that the virus probably originated from an insect host. Several picorna-like sequences have been recently reported in soybean thrips [47]. Revisiting 674 non-host de novo assemblies showed the presence of a small number of contigs from thrips. These results suggest that the picornavirus sequences originated from thrips, one of the most difficult pests to control in greenhouses.

Two large contigs representing two endosymbionts of mealybug, *Planococcus citri*, were found in library #20. Endogenous bacteria of mealybugs were not identified by the workflow described above. Large de novo contigs that did not align with either host sequences, or with the three in-house databases were identified by NCBI blast search as endogenous bacteria of mealybugs.

### 4.6. Pathogen Status of Citrus Relatives Held in Quarantine

Four citrus relatives (samples 1–4; Table 1) included in the present study have been maintained in the USDA Repository greenhouses for many years. Our NGS analysis as well as previous laboratory tests did not find any citrus viruses or known bacterial pathogens in all four accessions tested (Table 5). Since many citrus relatives are not graft-compatible with common indicator plants used in biological indexing, there is a need to develop alternate methods of testing before the release and distribution of germplasm. Some genera closely related to *Citrus*, such as *Microcitrus* and *Eremocitrus*, have been shown to have excellent tolerance/resistance to HLB [48], and have been used in breeding for HLB resistance [49]. There is a need to screen the hybrids for resistance in multiple geographic regions to assess their usefulness to the industry. Such germplasm exchanges would require approved, rapid, pathogen testing methods. We propose NGS transcriptome analysis as described here as an alternate method for releasing Rutaceous germplasm from quarantine as well as for exchanging breeders’ materials between industries. This would have to be approved by regulatory agencies, potentially at different levels (Federal and State in the USA).

### 4.7. Requirements for the Use of NGS as a Regulatory Test for Detection of Pathogens

For NGS methods to be used routinely, certain guidelines need to be developed and implemented. Protocols for nucleic acid extractions, guidelines for sequencing technologies recommended, minimum depth of sequencing, pipelines for different testing needs, etc. need to be standardized. The methods proposed should be user-friendly so that involved research groups can participate without requiring significant resources. The participating labs should be able to carry out the testing methods. Training sessions may be offered either by the regulatory agencies or laboratories that have successfully used the methods. NGS can serve as the main tool for testing germplasm in combination with other lab-based methods and biological indexing as optional confirmatory tests when needed. At the very least, NGS methods can be used along with the standard testing methods until proper evaluations are done.

### 4.8. Conclusions

Next-generation sequencing is a powerful technology that can be used as an additional tool for testing citrus germplasm. The movement of clean propagative materials across different regions requires quick and thorough testing of the germplasm. NGS would not only complement the currently used biological and laboratory assays but would also facilitate the detection of new pathogens as demonstrated in our present study. The introduction of budwood-carrying pathogens can be detrimental to the importing citrus industries if the pathogen spreads in the new region through insect vectors, farm tools, or other modes of dissemination. The addition of NGS methods to the routine disease testing regimen will complement the currently used testing methods and is beneficial for citrus germplasm certification and movement across state and international borders. Biological assays using standard citrus indicators are not amenable for testing non-citrus Rutaceous germplasm because of graft incompatibility. These citrus relatives are gaining importance because of their possible usefulness in developing resistance to HLB. NGS can be used as a reliable method for pathogen testing.

## Figures and Tables

**Table 1:** Details of 21 samples used for RNA-Seq analysis for identification of viral and bacterial pathogens. The description is based on information documented at the time of sample collection. Further details on most samples are available in the USDA ARS GRIN database. The inventory number is specific to the plant from which samples were collected for the study, and the accession number refers to a genotype or selection.

Library	Sample	Inventory (IVN0)	Accession	Description	Host	Bioproject Sample
1	1	7288	PI 358849	Held in quarantine; unknown disease status	*Clausena anisata*	SAMN18041798
2	2	1885	PI 600640	Held in quarantine; unknown disease status	*C. harmandiana*	SAMN18041799
3	3	9195	PI 539748	Held in quarantine; unknown disease status	*Hesperethusa crenulata*	SAMN18041800
4	4	9634	PI 539745	Held in quarantine; unknown disease status	*Bergera koenigii*	SAMN18041801
5 ^a^	5	4501	RPOS 7	Isolate E803 with viroids; Citrus bent leaf viroid, Hop stunt viroid, Citrus dwarfing viroid	*Citrus medica*	SAMN18041802
6	6	4488	RPOS 74	Isolate P215 with citrus psorosis virus	*C. sinensis*	SAMN18041803
7 ^a^	7	8078	RPOS 80	Isolate Cr1 with cristacortis, a disease of unknown etiology	*C. sinensis*	SAMN18041804
8	8	2530	RPOS 58	Isolate T 514 with citrus tristeza virus	*C. sinensis*	SAMN18041805
9	9	2091	RPOS 63	Isolate VE 703 with citrus vein enation virus	*C. sinensis*	SAMN18041806
10	10	NA ^c^	NA ^c^	*Citrus sinensis*, uninoculated seedling	*C. sinensis*	SAMN18041807
11	11	NA ^c^	NA ^c^	*Citrus sinensis*, with huanglonging- from a BSL3 facility	*C. sinensis*	SAMN18041808
12	12	9974	RPOS 84	Isolate C189 with *Spiroplasma citri*	*C. sinensis*	SAMN18041809
13	13	2025	RPOS 82	Isolate YV 920 with citrus yellow vein-associated virus	*C. medica*	SAMN18377111
14	14	4497	RPOS 83	Isolate FY 940, fatal yellows, a disease of unknown etiology	*C. macroptera*	SAMN18377112
15 ^b^	15	2032	RPOS 21	Isolate IV 402 with citrus variegation virus	*C. jambhiri*	SAMN18377113
	17	11,018	RPOS 112	Ilarvirus citrus leaf rugose virus, isolate CLRV 01	*C. sinensis*	
16 ^b^	16	2018	RPOS 19	Isolate IV 400 with citrus variegation virus	*C. medica*	SAMN18377114
	18	5046	RPOS 64	Citrus vein enation, isolate VE 704	*C. sinensis*	
17 ^a^	5	4501	RPOS 7	Isolate E803 with viroids; citrus bent leaf viroid, Hop stunt viroid, citrus dwarfing viroid	*C. medica*	SAMN18377115
18 ^a^	7	8078	RPOS 80	Isolate Cr1 with Cristacortis, a disease of unknown etiology	*C. sinensis*	SAMN18377116
19 ^b^	19	2327	RPOS 4	Isolate TL 101 with citrus tatter leaf virus	*C. jambhiri*	SAMN18377117
	21	3591	RRUT 178	‘Fuming Evergreen’ held in quarantine	*Poncirus trifoliata*	
20	20	7157	RPOS 79	Isolate CG P3K with concave gum, a disease of unknown etiology	*C. sinensis*	SAMN18377118

^a^ Sample 5 was used for constructing libraries 5 and 17, and Sample 7 was used for constructing libraries 7 and 18. ^b^ Libraries 15, 16, and 19 were constructed with mixtures containing two samples each. ^c^ Not applicable.

**Table 2:** Details of host sequence removal and de novo assembly of non-host sequences obtained from CLC Genomics Workbench analysis.

Library	Inventory (IVNO)	Number of Reads	Read Length	*C. clementina* ^a^	*C. sinensis* ^a^	Chloroplast ^a^	Mitochondria ^a^	Unmapped Reads	Percent	De Novo Contigs
1	7288	30,135,222	100	78.83	3.62	1.13	5.63	3,134,851	10.40	7491
2	1885	32,704,672	100	78.54	3.27	1.15	4.45	3,987,653	12.19	17,854
3	9195	28,877,614	100	74.83	6.25	1.82	7.49	646,974	2.24	990
4	9634	20,385,016	100	91.11	1.92	0.77	2.82	592,528	2.91	193
5	4501	51,047,772	100	85.77	4.46	0.71	7.28	720,868	1.41	1010
6	4488	34,345,866	100	79.50	6.77	2.30	6.77	4,556,457	13.27	674
7	8078	30,210,082	100	80.47	6.66	1.42	9.10	596,108	1.97	388
8	2530	26,975,152	100	85.03	4.08	0.99	5.65	1,019,877	3.78	193
9	2091	26,524,232	100	83.52	5.35	0.89	7.11	727,144	2.74	540
10	NA	25,852,318	100	79.71	6.32	1.90	7.95	963,629	3.73	347
11	NA	31,221,196	100	66.38	7.92	1.63	8.81	4,620,457	14.80	2168
12	9974	27,154,858	100	81.73	6.67	1.72	8.26	317,395	1.17	217
13	2025	120,761,450	100	84.45	4.00	0.73	5.82	6,038,470	7.50	3417
14	4497	130,559,826	100	75.29	10.51	4.29	8.34	2,056,638	2.36	1649
15	2032	115,838,044	150	72.27	5.60	0.77	7.48	16,068,989	20.81	2310
	11,018									
16	2018	125,407,824	150	78.98	7.57	0.68	8.65	5,159,223	6.17	2396
	5046									
17	4501	115,445,780	150	91.08	0.22	0.69	4.58	1,853,382	2.41	427
18	8078	116,612,916	150	81.11	8.11	0.80	8.64	1,564,171	2.01	4277
19	2327	98,699,626	150	74.91	9.45	0.91	12.37	2,323,833	3.53	2518
	3591									
20	7157	126,148,294	150	67.23	6.16	0.90	7.63	22,798,246	27.11	49,976

^a^ Host sequences were removed by mapping the sequence reads to the nuclear genomes of *Citrus clementina*, *C. sinensis*, and chloroplastic/mitochondrial sequences of *C. sinensis*. NA—Not applicable.

**Table 3:** Names and abbreviations of citrus pathogens detected in this study according to current and earlier nomenclatures.

Pathogen Names and Abbreviations	Alternate Names and Abbreviations
Citrus bent leaf viroid (CBLVd)	Citrus viroid I (CVd-I)
Hop stunt viroid (HSVd)	Citrus viroid II (CVd-II) Citrus cachexia viroid (CCaVd)
Citrus dwarfing viroid (CDVd)	Citrus viroid III (CVd-III)
Citrus bark cracking viroid (CBCVd)	Citrus viroid IV (CVd-IV)
Citrus viroid V (CVd-V)	
Citrus viroid VI (CVd-VI)	
Citrus exocortis viroid (CEVd)	
Citrus tristeza viroid (CTV)	
Citrus tatter leaf virus (CTLV)	
Citrus leaf rugose virus (CLRV)	
Citrus variegation virus (CVV)	Infectious variegation (IV)
Citrus vein enation virus (CVEV)	
Citrus leaf blotch virus (CLBV)	Dweet mottle virus (DMV)
Citrus psorosis virus (CPsV)	
Citrus virus A (CiVA)	
Beet western yellows virus (BWYV) ^a^	
Citrus yellow vein-associated virus (CYVaV)	
*Spiroplasma citri* (SC)	
*Candidatus* Liberibacter asiaticus (CLas)	
Citrus blight-associated pararetrovirus (CBaPRV) ^a^	

^a^ These are identified in some citrus samples, but there appears to be no evidence of their pathogenicity.

**Table 4:** Viruses and bacteria identified through NGS in the present study. Blast search of de novo assemblies of non-host sequence reads was done against the in-house databases of viruses and bacteria as described in materials and methods.

Library	Identifier	Reads Unmapped to Host	Pathogen Identified	Contig Length	Reference Accession	Reads Mapped	Pathogen Genome Coverage (x)
5	E803	720,868	Beet western yellows virus	5678	NC_004756	2312	41
5	E803	720,868	Citrus dwarfing viroid	276	MN379508	13,223	4861
5	E803	720,868	Citrus viroid V	294	MN885656	533	181
5	E803	720,868	Citrus viroid V	294	NC_010165	482	164
6	P215	4,556,457	Citrus dwarfing viroid	291	MF421250	1043	361
6	P215	4,556,457	Citrus hop stunt viroid	303	KY654683	791	265
6	P215	4,556,457	Citrus psorosis virus RNA1	8186	MG673944	12,235	149
6	P215	4,556,457	Citrus psorosis virus RNA2	1645	MG673945	14,010	852
6	P215	4,556,457	Citrus psorosis virus RNA2	1447	MG673946	7946	549
6	P215	4,556,457	Citrus virus A RNA1	6690	MZ436804	4896	73
6	P215	4,556,457	Citrus virus A RNA2	2735	MT922053	29,972	1094
7	Cristacortis	596,108	Citrus bark cracking viroid	206	KC121568	1129	548
7	Cristacortis	596,108	Citrus bent leaf viroid	327	MH200818	196	60
7	Cristacortis	596,108	Citrus cachexia viroid	301	AF213499	942	313
7	Cristacortis	596,108	Citrus dwarfing viroid	294	GQ254647	361	123
7	Cristacortis	596,108	Citrus psorosis virus RNA1	8186	AY654892	2202	27
7	Cristacortis	596,108	Citrus psorosis virus RNA2	1645	JN222364	3735	227
7	Cristacortis	596,108	Citrus psorosis virus RNA3	3101	AY654894	1447	100
8	T514	1,019,877	Citrus tristeza virus, T514	19,298	KC748391	197,602	1026
9	VE703	727,144	Citrus exocortis viroid	371	FJ904297	398	107
9	VE703	727,144	Citrus vein enation virus	5983	MN187037	143,613	2400
9	VE703	727,144	Hop stunt viroid	310	FJ716174	478	153
13	YV920	6,038,470	Citrus exocortis viroid	388	FJ751926	12,607	4787
13	YV920	6,038,470	Citrus leaf blotch virus	8729	EU857539	5080	87
13	YV920	6,038,470	Citrus variegation virus RNA1	3303	NC_009537	418	18
13	YV920	6,038,470	Citrus variegation virus RNA2	2592	NC_009538	269	14
13	YV920	6,038,470	Citrus variegation virus RNA3	2202	NC_009536	448	29
13	YV920	6,038,470	Citrus vein enation virus	5981	MN187041	575,045	14,417
13	YV920	6,038,470	Citrus yellow vein-associated virus	2692	MT893740	943,385	49,582
13	YV920	6,038,470	Hop stunt viroid	303	MT917190	209	103
14	FY940	2,056,638	Citrus leaf rugose virus RNA1	2834	NC_003548	130	6
14	FY940	2,056,638	Citrus leaf rugose virus RNA2	2676	NC_003547	148	7
14	FY940	2,056,638	Citrus leaf rugose virus RNA3	2107	NC_003546	329	22
14	FY940	2,056,638	Citrus variegation virus RNA1	3090	NC_009537	140	6
14	FY940	2,056,638	Citrus variegation virus RNA2	2599	NC_009538	239	12
14	FY940	2,056,638	Citrus variegation virus RNA3	2152	NC_009536	282	18
14	FY940	2,056,638	Citrus yellow vein-associated virus	2690	NC_040311	140,838	7848
15	IV402 & CLRV1	16,068,989	Citrus leaf rugose virus RNA1	2755	NC_003548	2,056,002	90,599
15	IV402 & CLRV1	16,068,989	Citrus leaf rugose virus RNA2	2940	NC_003547	2,508,070	125,823
15	IV402 & CLRV1	16,068,989	Citrus leaf rugose virus RNA3	1803	NC_003546	4,268,358	279,709
15	IV402 & CLRV1	16,068,989	Citrus variegation virus RNA1	2832	NC_009537	2,640,058	115,354
15	IV402 & CLRV1	16,068,989	Citrus variegation virus RNA2	2808	NC_009538	2,232,984	114,944
15	IV402 & CLRV1	16,068,989	Citrus variegation virus RNA3	2216	NC_009536	4,176,127	271,295
16	IV400 & VE704	5,159,223	Beet western yellow virus	5661	NC_004756	23,522	623
16	IV400 & VE704	5,159,223	Citrus dwarfing viroid	297	S76452	20,453	10,159
16	IV400 & VE704	5,159,223	Citrus exocortis viroid	371	MT917193	1325	533
16	IV400 & VE704	5,159,223	Citrus leaf rugose virus RNA1	3196	NC_003548	252,124	11,110
16	IV400 & VE704	5,159,223	Citrus leaf rugose virus RNA2	2749	NC_003547	188,179	9440
16	IV400 & VE704	5,159,223	Citrus leaf rugose virus RNA3	2161	NC_003546	85,273	5588
16	IV400 & VE704	5,159,223	Citrus variegation virus RNA1	3433	NC_009537	1,720,475	75,174
16	IV400 & VE704	5,159,223	Citrus variegation virus RNA2	2903	NC_009538	697,095	35,883
16	IV400 & VE704	5,159,223	Citrus variegation virus RNA3	2309	NC_009536	1,099,093	71,401
16	IV400 & VE704	5,159,223	Citrus vein enation virus	5983	NC_021564	395,231	9909
16	IV400 & VE704	5,159,223	Hop stunt viroid	296	NC_001351	11,888	5905
17	E803	1,853,382	Beet western yellows virus	5676	NC_004756	47,671	1260
17	E803	1,853,382	Citrus dwarfing viroid	271	MN379508	35,828	19,758
17	E803	1,853,382	Citrus variegation virus RNA1	3145	NC_009537	52	2
17	E803	1,853,382	Citrus variegation virus RNA2	1654	NC_009538	122	6
17	E803	1,853,382	Citrus variegation virus RNA3	2061	NC_009536	136	9
17	E803	1,853,382	Citrus viroid V	294	KY110720.1	126,634	64,830
17	E803	1,853,382	Citrus viroid V	280	JQ348925.1	57,735	29,457
18	Cristacortis	1,564,171	Citrus bark cracking viroid	206	KC121568	0	0
18	Cristacortis	1,564,171	Citrus bent leaf viroid	327	MH200818	9179	4211
18	Cristacortis	1,564,171	Citrus cachexia viroid	301	AF213499	14	7
18	Cristacortis	1,564,171	Citrus dwarfing viroid	294	GQ254647	4	2
18	Cristacortis	1,564,171	Citrus psorosis virus RNA1	8186	AY654892	9179	168
18	Cristacortis	1,564,171	Citrus psorosis virus RNA2	1645	AY654893	27,756	2531
18	Cristacortis	1,564,171	Citrus psorosis virus RNA3	1447	AY654894	36,236	3756
19	TL101 & RRUT 178	2,323,833	Citrus blight-associated pararetrovirus	7253	MN314438	13,112	271
19	TL101 & RRUT 178	2,323,833	Citrus tristeza virus	19,273	FJ525435	1,426,302	11,101
19	TL101 & RRUT 178	2,323,833	Citrus tatter leaf virus	6497	KC588948	6986	161
20	CG3PK	22,798,246	Citrus virus A RNA1	6691	MG764565	64,324	1442
20	CG3PK	22,798,246	Citrus virus A RNA2	2740	MG764566	148,928	8153

**Table 5:** Viruses and bacteria detected in 21 samples by NGS analysis and by other methods.

Library	Sample	Inventory (IVNO)	Description	Viruses and Bacteria Identified by NGS	Viruses and Bacteria Identified by Biological Indexing/qPCR/cPCR/Sequencing
1	1	7288	*Clausena anisata* held in quarantine; unknown disease status	none	none
2	2	1885	*Clausena harmandiana* held in quarantine; unknown disease status	none	none
3	3	9195	*Hesperethusa crenulata* held in quarantine; unknown disease status	none	none
4	4	9634	*Bergera koenigii* held in quarantine; unknown disease status	none	none
5	5	4501	Isolate E803 with viroids; citrus bent leaf viroid, Hop stunt viroid, citrus dwarfing viroid	BWYV, CDVd, CVd-V	BWYV ^b^, CVDd, CVd-V
6	6	4488	Isolate P215 with citrus psorosis virus	PLV ^a^, CDVd, HSVd, CiVA, CPsV	CVDd, HSVd, CiVA ^b^, CPsV
7	7	8078	Isolate Cr1 with Cristacortis, a disease of unknown etiology	CBCVd, CBLVd, CDVd, HSVd, CPsV	CBCVd, CBLVd, CDVd, HSVd, CPsV
8	8	2530	Isolate T 514 with Citrus tristeza virus	CTV	CTV
9	9	2091	Isolate VE 703 with Citrus vein enation virus	CVEV, HSVd, CEVd	CVEV, HSVd, CEVd
10	10		*Citrus sinensis,* uninoculated seedling	none	none
11	11		*Citrus sinensis*, with huanglonging- from a BSL3 facility	CLas	CLas
12	12	9974	Isolate C189 with *Spiroplasma citri*	SC	SC
13	13	2025	Isolate YV 920 with Citrus yellow vein-associated virus	CYVaV, HSVd, CEVd, CVEV, CVV ^d^	CYVaV ^b^, HSVd, CEVd, CVEV
14	14	4497	Isolate FY 940, fatal yellows, a disease of unknown etiology	CYVaV, CVV ^d^, CLRV ^d^,	CYVaV ^b^
15	15	2032	Isolate IV 402 with Citrus variegation virus	CVV, CDVd, CLRV	CVV, CDVd
	17	11,018	Ilarvirus Citrus leaf rugose virus		CLRV
16	16	2018	Isolate IV 400 with Citrus variegation virus	CVV, HSVd, CDVd, BWYV, CVEV, CEVd, CLRV ^d^	CVV, HSVd, CDVd, BWYV ^b^
	18	5046	Citrus vein enation, isolate VE 704		CEVd, CVEV
17	5	4501	Isolate E803 with viroids; Citrus bent leaf viroid, Hop stunt viroid, Citrus dwarfing viroid	BWYV, CDVd, CVd-V	BWYV ^b^, CDVd, CVd-V
18	7	8078	Isolate Cr1 with Cristacortis, a disease of unknown etiology	CBCVd, CBLVd, CDVd, HSVd, CPsV	CBCVd, CBLVd, CDVd, HSVd, CPsV
19	19	2327	Isolate TL 101 with citrus tatter leaf virus	CTLV, CTV, CBaPRV ^c^	CTLV
	21	3591	‘Fuming Evergreen’ held in quarantine		CTV, CBaPRV ^c^
20	20	7157	Isolate CG P3K with concave gum, a disease of unknown etiology	CiVA, *Ca*TP ^a^, *Ca*ME ^a^	CiVA ^b^

^a^ These sequences were determined to have originated from plant pests, thrips (PLV), and mealybugs (*Ca*TP & *Ca*ME). ^b^ Pathogen detection methods were developed in this study. ^c^ Our results indicate that CBaPRV sequences are endogenous in many citrus types and related taxa and are expressed highly in trifoliate and certain trifoliate hybrids (Section 2.6 below). ^d^ NGS analysis showed a very low population of these viruses, not detectable by other laboratory assays.

**Table 6:** Viral genome sequences deposited in the NCBI database from the present study.

Library	Virus Name	Isolate	Accession
5	Beet western yellows virus	E803/BWYV	MW674791
5	Citrus dwarfing viroid	E803/CDVd	MZ330073
5	Citrus viroid V	E803/CVd-V	MZ330081
6	Picorna-like virus	P215/PLV	MW674792
6	Citrus dwarfing viroid	P215/CDVd	MZ330074
6	Hop stunt viroid	P215/HSVd	MZ330075
6	Citrus virus A	P215/CiVA RNA1	MZ330076
6	Citrus virus A	P215/CiVA RNA2	MZ330077
6	Citrus psorosis virus	P215/CPsV RNA1	MZ330078
6	Citrus psorosis virus	P215/CPsV RNA2	MZ330079
6	Citrus psorosis virus	P215/CPsV RNA3	MZ330080
7	Citrus bark cracking viroid	citrus cristacortis/CBCVd	MZ330082
7	Citrus bent leaf viroid	citrus cristacortis/CBLVd	MZ330083
7	Citrus dwarfing viroid	citrus cristacortis/CDVd	MZ330084
7	Hop stunt viroid	citrus cristacortis/HSVd	MZ330085
7	Citrus psorosis virus	citrus cristacortis/CPsV RNA1	MZ330086
7	Citrus psorosis virus	citrus cristacortis/CPsV RNA2	MZ330087
7	Citrus psorosis virus	citrus cristacortis/CPsV RNA3	MZ330088
13	Citrus yellow vein-associated virus	YV920/CYVaV	MZ330089
13	Citrus exocortis viroid	YV 920/CEVd	MZ330090
13	Hop stunt viroid	YV 920/HSVd	MZ330091
13	Citrus variegation virus	YV 920/CVV RNA1	MZ330092
13	Citrus variegation virus	YV 920/CVV RNA2	MZ330093
13	Citrus variegation virus	YV 920/CVV RNA3	MZ330094
13	Citrus vein enation virus	YV 920/CVEV	MZ330095
14	Citrus yellow vein-associated virus	FY 940/CYVV	MZ330113
15	Citrus variegation virus	IV 402/CVV RNA1	MZ330096
15	Citrus variegation virus	IV 402/CVV RNA2	MZ330097
15	Citrus variegation virus	IV 402/CVV RNA3	MZ330098
15	Citrus dwarfing viroid	IV 402/CDVd	MZ330099
15	Citrus leaf rugose virus	IV 402/CLRV RNA1	MZ330100
15	Citrus leaf rugose virus	IV 402/CLRV RNA2	MZ330101
15	Citrus leaf rugose virus	IV 402/CVV RNA2	MZ330102
15	Hop stunt viroid	IV 400/HSVd	MZ330105
16	Citrus dwarfing viroid	IV 400/CDVd	MZ330106
16	Citrus exocortis viroid	VE 704/CEVd	MZ330107
16	Beet western yellows virus	IV 400/BWYV	MZ330108
16	Citrus variegation virus	IV 400/CVV RNA1	MZ330109
16	Citrus variegation virus	IV 400/CVV RNA2	MZ330110
16	Citrus variegation virus	IV 400/CVV RNA3	MZ330111
16	Citrus vein enation virus	VE 704/CVEV	MZ330112
19	Citrus blight-associated pararetrovirus	RRUT 178/CBaPRV	MZ330114
19	Citrus tatter leaf virus	TL 101/CTLV	MZ330115
19	Citrus tristeza virus	RRUT 178/CTV	MZ330116
20	Citrus virus A	CG P3K/CiVA RNA1	MZ330103
20	Citrus virus A	CG P3K/CiVA RNA2	MZ330104

**Table 7:** A comparison of genomic regions of three different isolates of citrus yellow vein-associated virus (CYVaV) isolates. The sequence of YV920a (JX101610.1) was taken from a previous study [19], and sequences of YV920b (MZ330089.1) and FY940 (MZ30113.1) are from the present study.

Genome Regions	YV920a	YV920b	FY940
Whole genome (nt ^a^)	100	98	86
5’ untranslated (nt)	100	100	100
RdRP (aa ^b^)	100	98	91
Hypothetical protein (aa)	100	96	86
3’ UTR (nt)	100	98	86

^a^ Percent nucleotide (nt) identity; ^b^ Percent amino acid identity.

**Table 8:** Results of standard curve analysis of primers and probes by real-time PCR using serial dilutions of the corresponding templates constructed by synthetic gBlocks^TM^.

Targets	y	R^2^	Efficiency%
BWYV	−3.4299x + 40.822	0.9984	95.718
CYVaV	−3.4682x + 38.22	0.9988	94.245
CBaPRV	−3.4530x + 37.041	0.9986	94.806
CiVA	−3.5195x + 38.348	0.9981	92.385
MDH_RNA	−3.5472x + 40.578	0.9972	91.394

**Table 9:** Testing plants from the positive plant inventory for four viruses of interest by real-time PCR assays. A total of 115 accessions were tested. Only plants positive for BWYV, CYVaV, and CiVA are shown.

Accession	Inventory	Database Description	Virus Detected	Other Viruses Detected ^b^	Origin	Comment ^c^
RPOS 7	4501 ^a^	CVd Ia + CVd IIa + CVd IIIb (E 803)	BWYV	CDVd, CVd-V	California	Collected from ‘Frost nucellar’ navel in 1958 as a mixture of CVd I, II and III. In later years, viroids I was reported to have disappeared.
RPOS 8	5085	CVd III (Lake Alfred)	BWYV	HSVd, CDVd	Florida	From CREC, Lake Alfred.
RPOS 10	5087	CVD IIb (Ca 902)	BWYV	HSVd, CVd-V	California	Collected in 1963 from old line navel orange.
RPOS 11	5080	CVd IIb (Ca 907)	BWYV	HSVd, CEVd	Spain	Isolated from ‘Ricote’ lemon. Mild positive for Cachexia, used as control in bioindexing. CEVd, CVds IIb and IIIa (reported by Semancik)
RPOS 19	2018	IV 400	BWYV	HSVd, CVV	California	Collected from ‘Eureka’ lemon in Davey Grove in 60s.
RPOS 82	2025 ^a^	YV 920	BWYV & CYVaV	HSVd, CEVd, CVEV	California	Collected by Wallace in 1957 from Tulare County.
RPOS 83	4497 ^a^	FY 940	CYVaV	None	California	Introduced by Schneider in 1980 from ‘Eureka’ lemon trees on *C. macrophylla*
RPOS 13	5102 ^a^	CG 301	CiVA	None	California	Collected from UC Riverside fields in the 60s.
RPOS 73	4489	P 214	CiVA	None	California	Collected in 1982 from variegated sweet orange. Identified putatively as psorosis, but suspected as a new virus.
RPOS 74	4488 ^a^	P 215	CiVA	HSVd, CDVd, CPsV, CiVA	California	Collected in 1980 from Redlands, CA. Very strong reaction on indicators, mechanically transmissible to citrons and *Chenopodium*.
RPOS 78	2326	CG 307	CiVA	none	California	From block 14 of UC Riverside, collected in 1983 from a tree with concavities on the trunk.
RPOS 79	7157 ^a^	CG P3K	CiVA	none	Florida	Received from Steve Garnsey, USDA Horticultural Research Laboratory, Orlando, Florida.
RPOS 103	11,207	CG 302	CiVA	HSVd	California	Collected by Drake in 1962 from a navel orange from Rancho Santa Ana.

^a^ Also included in the analysis by NGS transcriptome analysis in the present study (see Table 5). ^b^ Other pathogens were detected previously by qPCR, conventional PCR, Sanger sequencing, and/or bioindexing. ^c^ Based on information from local and USDA-GRIN databases.

**Table 10:** Testing citrus germplasm accessions for the citrus blight-associated pararetrovirus gene expression by qPCR.

				Ct Values		
Citrus Group	Total	Negative	Positive	Minimum	Maximum	Average
Citrandin	2	0	2	23	24	23.50
Trifoliate	59	2	57	19	23	24.03
Trifoliate hybrid	22	0	22	21	34	25.35
Citrange	16	0	16	20	33	25.96
Citrumelo	9	0	9	21	32	26.24
Kumquat hybrid *	17	10	7	25	29	26.60
Tangelo	29	28	1	28	28	28.00
Pummelo	72	70	2	35	36	35.62
Lemon	143	139	4	33	39	36.04
Mandarin & hybrid	110	104	6	35	39	36.21
Citron	130	126	4	36	37	36.40
Papeda	40	39	1	37	37	37.47
Citrus relative	24	22	0			
Grapefruit	38	38	0			
Grapefruit hybrid	10	10	0			
Kumquat	4	4	0			
Lemon hybrid	29	29	0			
Lime	47	47	0			
Lime hybrid	6	6	0			
Microcitrus & hybrid	8	8	0			
Navel orange	40	40	0			
Rangpur	7	7	0			
Rough lemon	20	20	0			
Sour orange & hybrid	47	47	0			
Sweet lime and lemon	13	13	0			
Sweet orange	95	95	0			
Tangor	25	25	0			
Total	1062	929	131			

* hybrids with trifoliate parentage may show positive reaction for the citrus blight-associated pararetrovirus.

## Data Availability

Information on accessions used in this study is available from the National Plant Germplasm System, and raw sequence reads can be accessed by accessing the relevant Bioproject sample in Table 1 on NCBI. Total sequence reads, and depth of coverage of different viral genomes from 20 plant samples are shown in Table 4, and 46 full-length genomes of viruses (Table 6) are available from NCBI.

## Supplementary Materials

The following supporting information can be downloaded at: https://www.mdpi.com/article/10.3390/plants13030411/s1, Figure S1: Phylogeny of Beet western yellows virus (BWYV). Genomes from different geographic locations in different host plants were analyzed. Pea enation mosaic virus-1 (PEMV-1, NC_003629) was used as an outgroup sequence. Accession numbers of different BWYV genomes, plant host information, and geographic locations are shown. The phylogenetic tree was constructed using the neighbor-joining method and visualized with MEGA11.0. Bootstrap values shown at the nodes indicate the percentage of 1000 replications supporting the clades; Figure S2: Alignment of DNA and RNA of the target area selected for designing primers and probe for real-time PCR amplification of reverse-transcribed RNA. The RNA sequence shown constitutes the 3’ terminus of exon 12 and 5’ end of exon 13 of the malate dehydrogenase gene. The forward primer was designed to align mostly to the 3’ end of exon 12 and three bases at the 5’ terminus of exon 13 so that the amplification is restricted only to reverse-transcribed RNA. The sequences of the probe and the primers as well as the direction are shown. Intron sequences are indicated by dots in the corresponding regions; Figure S3: Alignment of the target region of Citrus blight-associated pararetrovirus (MZ330114) selected for real-time PCR assay. CBaPRV reported to be associated with citrus blight (MN14438.1), and from the genomes of *Poncirus trifoliata*, *Citrus sinensis*, *C. clementina*, *Eremocitrus glauca* and *Atalantia buxifolia*. The primers and probe region are highlighted, and their directions of synthesis are shown. Multiple copies of full-length or near full-length genomes of CBaPRV are in all the genomes shown in the diagram; Table S1: Percent sequence identity matrix between Beet western yellows virus isolates from citrus and other closely related viruses. The sequences of 20 isolates of BWYV were downloaded from NCBI Genbank. The sequences were aligned using ClustalW (reference) to generate the phylogenetic tree (Figure 2); Table S2: Location of full-length genome sequence of CBaPRV in different chromosomes of the five selected taxa of the tribe Citreae, subfamily Aurantioideae. Only one copy with the highest nucleotide identity is shown for each chromosome. Multiple copies were found in almost all chromosomes. Query coverage and percent identity with the CBaPRV (MZ330114) identified in the present study are shown.
