# Supplementary material for: Next Generation Sequencing, and Development of a Pipeline as a Tool for the Detection and Discovery of Citrus Pathogens to Facilitate Safer Germplasm Exchange
Source: Plants (Basel). 2024 Jan 30;13(3):411. doi: 10.3390/plants13030411 (PMC10856814; doi:10.3390/plants13030411)
Supplement: Supplementary file 1 [file plants-13-00411-s001.zip › plants-2822034-supplementary.pdf]

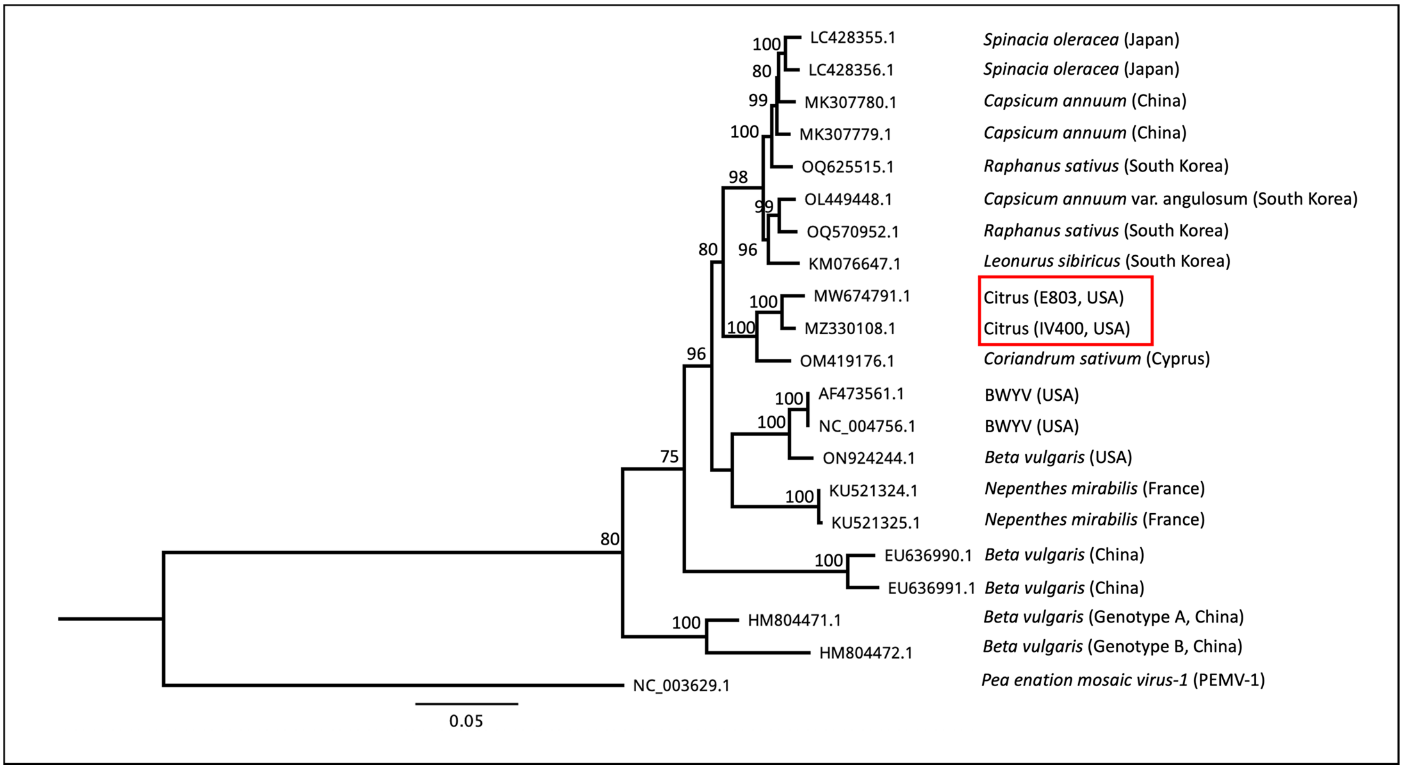

Figure S1. Phylogeny of Beet western yellows virus (BWYV). Genomes from different geographic locations in different host plants were analyzed. Pea enation mosaic virus-1 (PEMV-1, NC_003629) was used as an outgroup sequence. Accession numbers of different BWYV genomes, plant host information, and geographic locations are shown. The phylogenetic tree was constructed using the neighbor-joining method and visualized with MEGA11.0. Bootstrap values shown at the nodes indicate the percentage of 1000 replications supporting the clades.

```
DNA        ATATGTCTTGTAGACTCAAAGGTAGTAGAAGGCTTGATTGAGATTCTTTGGCGACCAAAAAGTTTATCTG
RNA        ATATGTCTTGTAGACTCAAAGG::::::::::::::::::::::::::::::::::::::::::::::::
PRIMER     ATATGTCTTGTAGACTCAAAGG::::::::::::::::::::::::::::::::::::::::::::::::
              FORWARD →

DNA        CTAGTCTCTGTATTCATTTCTTATTTTGCATGCAGGGACTGATAGTCAGCTCCCGGAAAGATTCACTTCA
RNA        ::::::::::::::::::::::::::::::::::::GACTGATAGTCAGCTCCCGGAAAGATTCACTTCA
PRIMER     ::::::::::::::::::::::::::::::::::::GAC
                                                →

DNA        ACACTTCAAGAAGCCTTGGGCTCATGAACATGAGCCTGTCAACAATC
RNA        ACACTTCAAGAAGCCTTGGGCTCATGAACATGAGCCTGTCAACAATC
PRIMER     ACACTTCAAGAAGCCTTGGGCTCA GAACATGAGCCTGTCAACAATC
               PROBE →                     ← REVERSE
```

Figure S2. Alignment of DNA and RNA of the target area selected for designing primers and probe for real-time PCR amplification of reverse-transcribed RNA. The RNA sequence shown constitutes the 3′ terminus of exon 12 and 5′ end of exon 13 of the malate dehydrogenase gene. The forward primer was designed to align mostly to the 3′ end of exon 12 and three bases at the 5′ terminus of exon 13 so that the amplification is restricted only to reverse-transcribed RNA. The sequences of the probe and the primers as well as the direction are shown. Intron sequences are indicated by dots in the corresponding regions.

```
                           Forward →                                    Probe →                  ← Reverse
Target sequence    ACCTTGATCGTATGCCATTACCTGAAGCTCAAATGTGGACATCCGTGTTCATCATTCATCGACCTTATTTCCAACACCCAGATACACAGTTCT
MZ330114.1         .............................................................................................
MN814438.1         .............................................................................................
P. trifoliata      .......A..........CCT.C.....A..............T........T..C..CA.G..............T........C..TC...
C. sinenis         ................TACC........A.......TC.....A..T..T..T.....CA....A..C..T.....T.....C..CA.AC...
C. clementina      .......C........TGCC........A.......TC........T..T..T.....CA.G..A..C........T.....C..C..TC...
E. glauca          .T.....A........TG.CT.A.....A...C...TT..C..A..A........C...A.G..A..C...T.G..T.TCT.....A.TC.T.
A. buxifolia       .TT.G..A..G......CC...C.....AA......TC..............T....G...G..A..C........T.....C..-...C...
```

Figure S3. Alignment of the target region of Citrus blight-associated pararetrovirus (MZ330114) selected for real-time PCR assay. CBaPRV reported to be associated with citrus blight (MN14438.1), and from the genomes of *Poncirus trifoliata*, *Citrus sinensis*, *C. clementina*, *Eremocitrus glauca* and *Atalantia buxifolia*. The primers and probe region are highlighted, and their directions of synthesis are shown. Multiple copies of full-length or near full-length genomes of CBaPRV are in all the genomes shown in the diagram.

Table S1. Percent sequence identity matrix between Beet western yellows virus isolates from citrus and other closely related viruses. The sequences of 20 isolates of BWYV were downloaded from NCBI Genbank. The sequences were aligned using ClustalW (reference) to generate the phylogenetic tree (Figure 2).

**No.**	**Host**	**Location**	**Accession**	**1**	**2**	**3**	**4**	**5**	**6**	**7**	**8**	**9**	**10**	**11**	**12**	**13**	**14**	**15**	**16**	**17**	**18**	**19**	**20**	**21**
1	*Beta vulgaris*	USA	AF473561.1		85	85	84	82	91	92	92	91	91	92	91	91	92	48	100	91	92	98	91	91
2	*Beta vulgaris*	China	EU636990.1	85		97	78	79	85	85	84	85	85	86	85	85	85	46	85	86	85	85	85	86
3	*Beta vulgaris*	China	EU636991.1	85	97		78	78	85	85	85	85	85	85	85	85	85	44	85	85	85	85	85	85
4	*Beta vulgaris*	China	HM804471.1	84	78	78		94	88	83	84	88	88	88	88	85	85	48	84	87	87	83	88	87
5	*Beta vulgaris*	China	HM804472.1	82	79	78	94		83	81	82	83	83	83	83	81	81	48	82	83	81	81	83	83
6	*Leonurus sibiricus*	S. Korea	KM076647.1	91	85	85	88	83		91	91	97	97	97	97	92	93	44	91	98	93	91	97	97
7	*Nepenthes mirabilis*	France	KU521324.1	92	85	85	83	81	91		100	91	91	91	91	91	92	45	92	91	92	92	91	91
8	*Nepenthes mirabilis*	France	KU521325.1	92	84	85	84	82	91	100		90	91	91	91	91	91	45	92	90	92	92	90	91
9	*Spinacea oleracea*	Japan	LC428355.1	91	85	85	88	83	97	91	90		99	98	98	93	93	46	91	97	93	91	97	98
10	*Spinacea oleracea*	Japan	LC428356.1	91	85	85	88	83	97	91	91	99		98	98	93	93	47	91	97	93	91	97	98
11	*Capsicum annuum*	China	MK307779.1	92	86	85	88	83	97	91	91	98	98		99	93	93	46	92	97	94	91	97	98
12	*Capsicum annuum*	China	MK307780.1	91	85	85	88	83	97	91	91	98	98	99		93	93	46	91	97	94	91	97	98
13	*Citrus medica*	USA	MW674791.1	91	85	85	85	81	92	91	91	93	93	93	93		98	45	91	93	96	91	93	93
14	*Citrus medica*	USA	MZ330108.1	92	85	85	85	81	93	92	91	93	93	93	93	98		48	92	93	97	92	93	93
15	*Pisum sativum*	USA	NC_003629.1	48	46	44	48	48	44	45	45	46	47	46	46	45	48		48	47	48	46	48	46
16	*Beta vulgaris*	USA	NC_004756.1	100	85	85	84	82	91	92	92	91	91	92	91	91	92	48		91	92	98	91	91
17	*Capsicum annuum*	S. Korea	OL449448.1	91	86	85	87	83	98	91	90	97	97	97	97	93	93	47	91		93	91	98	98
18	*Coriandrum sativum*	Cyprus	OM419176.1	92	85	85	87	81	93	92	92	93	93	94	94	96	97	48	92	93		92	93	93
19	*Beta vulgaris*	USA	ON924244.1	98	85	85	83	81	91	92	92	91	91	91	91	91	92	46	98	91	92		91	91

20	*Raphanus sativus*	S. Korea	OQ570952.1	91	85	85	88	83	97	91	90	97	97	97	97	93	93	48	91	98	93	91		97
21	*Raphanus sativus*	S. Korea	OQ625515.1	91	86	85	87	83	97	91	91	98	98	98	98	93	93	46	91	98	93	91	97	

Table S2. Location of full-length genome sequence of CBaPRV in different chromosomes of the five selected taxa of the tribe Citreae, subfamily Aurantioideae. Only one copy with the highest nucleotide identity is shown for each chromosome. Multiple copies were found in almost all chromosomes. Query coverage and percent identity with the CBaPRV (MZ330114) identified in the present study are shown.

Species	Chromosome	Sequence ID	Range from	Range to	Query coverage	Percent identity
Poncirus trifoliata	1	CM031384.1	15,258,238	15,265,473	100	90
Poncirus trifoliata	2	CM031385.1	14,284,603	14,291,729	100	90
Poncirus trifoliata	3	CM031386.1	19,006,443	19,013,566	100	90
Poncirus trifoliata	4	CM031387.1	13,956,427	13,963,543	100	90
Poncirus trifoliata	5	CM031388.1	26,910,377	26,917,490	100	90
Poncirus trifoliata	6	CM031388.1	26,910,377	26,917,490	100	90
Poncirus trifoliata	7	CM031390.1	11,764,897	11,770,635	100	90
Poncirus trifoliata	8					
Poncirus trifoliata	9	CM031390.1	11,764,897	11,770,635	100	90
Citrus sinensis	1	NC_068556.1	7,919,834	7,926,944	98	89
Citrus sinensis	2	NC_068557.1	16,315,933	16,322,504	98	89
Citrus sinensis	3	NC_068558.1	38,342,981	38,350,083	98	89
Citrus sinensis	4	NC_068559.1	17,935,299	17,942,409	99	88
Citrus sinensis	5	NC_068560.1	22,316,558	22,323,239	98	89
Citrus sinensis	6	NC_068561.1	1,144,084	1,151,187	98	89
Citrus sinensis	7	NC_068562.1	18,773,731	18,780,838	98	89
Citrus sinensis	8	NC_068563.1	10,090,659	10,097,745	98	88
Citrus sinensis	9	NC_068564.1	14,567,213	14,574,311	98	87
Citrus clementina	1	NW_006263303.1	7,637,029	7,644,138	98	89
Citrus clementina	2	NW_006262688.1	19,946,972	19,954,060	99	88
Citrus clementina	3	NW_006262339.1	47,529,358	47,536,447	98	88
Citrus clementina	4	NW_006262274.1	16,401,624	16,408,153	98	89
Citrus clementina	5	NW_006262201.1	12,443,578	12,449,639	98	90
Citrus clementina	6	NW_006262139.1	1,916,953	1,923,982	98	89
Citrus clementina	7	NW_006262075.1	9,687,551	9,690,677	88	90
Citrus clementina	8	NW_006262022.1	1,998,592	2,005,694	99	87
Citrus clementina	9	NW_006261964.1	18,517,351	18,524,443	98	87
Eremocitrus glauca	1	JAQOOD010000001.1	20,013,605	20,019,870	93	87
Eremocitrus glauca	2	JAQOOD010000002.1	1,594,053	1,600,331	96	87
Eremocitrus glauca	3	JAQOOD010000003.1	27824048	27,830,330	95	86
Eremocitrus glauca	4	JAQOOD010000004.1	15,795,358	15,801,629	95	87
Eremocitrus glauca	5	JAQOOD010000005.1	16,384,809	16,391,080	95	87

Eremocitrus glauca	6	JAQOOD010000006.1	7901417	7,907,674	95	86
Eremocitrus glauca	7	JAQOOD010000007.1	19,260,724	19,266,917	96	87
Eremocitrus glauca	8	JAQOOD010000008.1	17,441,403	17,447,672	97	87
Eremocitrus glauca	9	JAQOOD010000009.1	16,812,589	16,818,875	98	87
Atalantia buxifolia	1	CM059831.1	24,156,300	24,162,430	95	83
Atalantia buxifolia	2	CM059832.1	15,464,010	15,470,179	94	83
Atalantia buxifolia	3	CM059833.1	12,092,001	1,209,805	95	84
Atalantia buxifolia	4	CM059834.1	6,994,663	7,000,836	95	83
Atalantia buxifolia	5	CM059835.1	30,036,681	30,042,827	94	83
Atalantia buxifolia	6	CM059836.1	17,147,129	17,153,277	94	83
Atalantia buxifolia	7	CM059837.1	10,911,282	10,917,334	95	83
Atalantia buxifolia	8	CM059838.1	3,262,858	3,269,002	95	83
Atalantia buxifolia	9	CM059839.1	16,715,379	16,721,473	95	83
